# Management of Patients With Acute Cholecystitis After Percutaneous Cholecystostomy: From the Acute Stage to Definitive Surgical Treatment

**DOI:** 10.3389/fsurg.2021.616320

**Published:** 2021-04-15

**Authors:** Yu-Liang Hung, Chang-Mu Sung, Chih-Yuan Fu, Chien-Hung Liao, Shang-Yu Wang, Jun-Te Hsu, Ta-Sen Yeh, Chun-Nan Yeh, Yi-Yin Jan

**Affiliations:** ^1^Division of General Surgery, Chang Gung Memorial Hospital, Taoyuan, Taiwan; ^2^Department of Gastroenterology and Hepatology, Chang Gung Memorial Hospital, Taoyuan, Taiwan; ^3^Division of Trauma and Emergency Surgery, Chang Gung Memorial Hospital, Taoyuan, Taiwan; ^4^College of Medicine, Chang Gung University, Taoyuan, Taiwan

**Keywords:** percutaneous cholecystostomy, percutaneous transhepatic gallbladder drainage, cholecystitis, cholecystectomy, cholangiogram

## Abstract

Percutaneous cholecystostomy (PC) has become an important procedure for the treatment of acute cholecystitis (AC). PC is currently applied for patients who cannot undergo immediate laparoscopic cholecystectomy. However, the management following PC has not been well-reviewed. The efficacy of PC tubes has already been indicated, and compared to complications of other invasive biliary procedures, complications related to PC are rare. Following the resolution of AC, patients who can tolerate anesthesia and the surgical risk should undergo interval cholecystectomy to reduce the recurrence of biliary events. For patients unfit for surgery, whether owing to comorbidities, anesthesia risks, or surgical risks, expectant management may be applied; however, a high incidence of recurrence has been noted. In addition, several interesting issues, such as the indications for cholangiography via the PC tube, removal or maintenance of the PC catheter before definitive treatment, and timing of elective surgery, are all discussed in this review, and a relevant decision-making flowchart is proposed. PC is an effective and safe intervention, whether as expectant treatment or bridge therapy to definitive surgery. High-level evidence of post-PC care is still necessary to modify current practices.

## Introduction

Percutaneous cholecystostomy (PC), usually performed by interventional radiologists, is an effective intervention to decompress the gallbladder in patients with acute cholecystitis (AC). The form of PC varies with the intervention approach and the placement of drainage catheters and includes percutaneous transhepatic gallbladder drainage (PTGBD), percutaneous transperitoneal gallbladder drainage, and percutaneous transhepatic gallbladder aspiration. The development of PC can be traced back to the 1970s, and PC was first applied in patients with obstructive jaundice (1). In the 1980s, PC was gradually conducted in patients with AC (2–7). Currently, PC is commonly indicated for patients with AC who are not suitable for immediate laparoscopic cholecystectomy (LC), such as those with severe sepsis, shock, or multiple comorbidities (8). The World Society of Emergency Surgery (WSES) guidelines, which are mostly applied in Western countries, have suggested that PC could be an alternative for patients unfit for emergency cholecystectomy due to the presence of severe comorbidities (9). On the other hand, the Tokyo guidelines, mostly applied in Eastern countries, recommended that both moderate AC patients who failed conservative treatment and severe AC patients with a high Charlson comorbidity index (CCI) and American Society of Anesthesiologists physical status (ASA-PS) should consider undergoing PC (10). PC is currently considered an alternative treatment for AC patients considered high risk for immediate surgery and can provide temporary relief from inflammation or infection resistant to conservative treatment in AC patients. However, the management and outcomes of AC patients after PC have not been well-reviewed. In addition, the importance of post-PC management has yet been emphasized in clinical guidelines, including Tokyo guidelines and WSES guidelines (9, 10). Therefore, in this review, we aimed to clarify the decision-making process and clinical outcomes of AC patients following PC.

## Methods

A relevant literature search was conducted in the MEDLINE, Embase, and Google Scholar databases. The databases were electronically searched from inception to April 24, 2020. For the MEDLINE database, the MeSH terms “Cholecystostomy,” “Cholecystitis,” and “Cholecystectomy” were utilized for the search. We also searched the keywords “PTGBD,” “Percutaneous transhepatic gallbladder drainage,” and “Percutaneous transperitoneal gallbladder drainage” in the form of free text typing. For the Embase database, the Emtree terms “Cholecystostomy,” “PTGBD,” “Percutaneous transhepatic gallbladder drainage,” “Percutaneous transperitoneal gallbladder drainage,” “Cholecystitis,” and “Cholecystectomy” were utilized for the search. For Google Scholar, “Cholecystostomy,” “PTGBD,” “Percutaneous transhepatic gallbladder drainage,” “Percutaneous transperitoneal gallbladder drainage,” “Cholecystitis,” and “Cholecystectomy” were utilized for the search. The literature search for journal articles was performed by a single author, and the applicability of the journal articles was judged by several experts in the field of biliary disease. A total of 79 publications relevant to the topic of this narrative review were identified by applying this strategy.

## Percutaneous Cholecystostomy Techniques: Transhepatic vs. Transperitoneal

Generally, there are two approaches for PC: transhepatic and transperitoneal. The transhepatic approach for PC, also known as PTGBD, is more common than the transperitoneal approach (11–14). Some authors prefer the transhepatic route over the transperitoneal route because previous studies revealed a higher incidence of complications, such as bile leakage and recurrence of cholecystitis, with the transperitoneal route (12, 15). However, recent publications have demonstrated that there are no significant differences in procedure-related complications or clinical outcomes between approaches (11, 16). The advantage of the transhepatic route is that this approach may provide more anatomic fixation than the transperitoneal route since the transhepatic route directly penetrates the liver. In addition, Hatjidakis et al. demonstrated that only ~2 weeks were required to develop a mature tract after transhepatic PC, which is a significantly shorter duration than that needed after transperitoneal PC (>3 weeks) (17). The advantage of the transperitoneal approach is that this route may benefit patients with distended gallbladders that directly adhere to the abdominal wall, coagulopathy, or liver disease since this approach does not penetrate the liver (15, 18). Technical difficulties are the main issue of the transperitoneal route, and a previous study indicated that <20% of patients were suitable for transperitoneal PC since the right colon might interpose into the space between the skin and gallbladder (19). In conclusion, transhepatic PC is more common, but the debate between transhepatic and transperitoneal PC remains ongoing. The choice of route can vary between individuals and radiologists but, most importantly, should depend on the anatomical structures and systemic condition of the patient.

## Efficacy of Percutaneous Cholecystostomy

PC is a technically feasible and safe alternative to emergent cholecystectomy for AC patients with multiple comorbidities or severe inflammation and infection (20–26). Winbladh et al. conducted a systematic review and demonstrated that up to 85.6% of PC procedures were successfully performed in more than 1,700 AC patients (27). PC is also recognized as an effective procedure that can drain infectious bile and decompress the gallbladder; furthermore, PC de-escalates the severity of systemic infection in a short time (20–22, 28–31). Regarding the duration from PC insertion to disease resolution, Noh et al. and Viste et al. indicated that most patients show clinical improvement in a median of 3–4 days after PC insertion (20, 32, 33). Moreover, Chou et al. revealed that performing early PC (<24 h) when AC is identified may benefit patients by shortening the hospital stay and reducing the incidence of procedure-related bleeding (30). Bickel et al. also revealed that patients with early PC insertion (<2 days) had a significantly lower incidence of conversing to open cholecystectomy followed by LC than those with late PC insertion (3–6 days) (34). Both Noh et al. and Viste et al. revealed significant decreases in white blood cell (WBC) and C-reactive protein (CRP) levels after PC; notably, the latter study mainly focused on patients with acute acalculous cholecystitis (32, 33). In addition, Chang et al. demonstrated short-term and long-term improvement on imaging after PC insertion, as evaluated by their newly proposed grading system (29). In brief, PC is a safe and effective alternative to cholecystectomy for AC patients with multiple comorbidities. Patients can benefit from PC in terms of clinical progress, radiographic improvement, and surgical outcomes of future cholecystectomy.

## Subsequent Management After Percutaneous Cholecystostomy

In previous studies, 60–70% of AC patients underwent PC without later interval LC (5, 12, 14, 15, 20, 27, 28, 31, 33, 35). A survey from a nationwide database conducted by Pavurala et al. also demonstrated that 62.2% of AC patients who underwent PC did not undergo interval cholecystectomy (36). Nevertheless, several studies have reported that a high incidence of biliary events, 22–41%, occurred during the 2.2–5 years follow-up period (12, 15, 20, 37). Some authors observed a high incidence of mortality among AC patients who underwent PC placement during the follow-up window. Interestingly, few of these patients died from biliary diseases; rather, most of them died from underlying non-biliary medical conditions (20, 24, 32, 33, 35, 38). Therefore, mortality, which is irrelevant to biliary disease, may be a competing factor for the recurrence of biliary events. Our previous study concluded that the recurrence of biliary events may be underestimated if mortality related to non-biliary events is not considered (37). The pathophysiology of recurrent biliary events has been proposed, and a possible mechanism may be related to impaired motility of the gallbladder after cholecystitis, the stasis of bile, and cystic duct obstruction (39–41). In addition, an animal experiment revealed that biofilms on PC tubes could also contribute to another episode of cholecystitis (42, 43). The clinical factors that could impact the recurrence of biliary disease have also been investigated, such as complicated cholecystitis, elevated CRP, and duration of PC catheter maintenance (28, 37, 44, 45). All of this evidence supports interval cholecystectomy as the first choice after PC placement if the patients can tolerate surgery.

### Acute Cholecystitis Patients Undergoing Percutaneous Cholecystostomy With Interval Cholecystectomy

Subsequent cholecystectomy following PC drainage is a safe and effective combined management strategy in AC patients (46–49). The rationale is that PC can rapidly de-escalate the inflammation and infection status of cholecystitis; after patients are medically optimized, removal of the gallbladder can prevent the recurrence of biliary events in the future (31, 49). Several studies have demonstrated that LC following PC can be performed safely with a small amount of intraoperative blood loss, a low incidence of conversion to open cholecystectomy (2.6–8%), and a low incidence of perioperative complications (5.3–8.6%) (47, 48, 50–53). Ke et al. also demonstrated a short duration of postoperative abdominal drainage (3.4 ± 2.1 days) and a low incidence of postoperative ICU admission (2%) (52). The mortality rate of LC following PC is extremely low, and one systemic review conducted by Winbladh et al. even reported a mortality rate of only 0.96% (5/523) (27, 51). Regarding expenses, a recent study based on the National Health Insurance Research Database (NHIRD) in Taiwan found that the average total medical expenses for AC patients with PC only, PC followed by cholecystectomy after 2 months, and PC followed by cholecystectomy within 2 months were 243,114, 190,970, and 172,370 NT$, respectively (54). Furthermore, with respect to medical expenses for recurrent biliary events, AC patients with PC only had 1.75 times higher expenses than AC patients with PC followed by cholecystectomy within 2 months (120,707 vs. 68,561 NT$) (54).

The currently published studies regarding the optimal interval from PC to cholecystectomy are summarized in Table 1 (48, 55–59). None of these studies were conducted prospectively, and the results were heterogeneous. Recently, Altieri et al. conducted a large-scale analysis based on the Statewide Planning and Research Collaborative System (SPARCS) database of New York State, which revealed that a duration ≤ 8 weeks (*n* = 1,211) was associated with a higher overall rate of complications and longer length of hospital stay than a duration > 8 weeks (*n* = 1,787) (59). However, this study still had some limitations, including a lack of information regarding the severity of AC, comorbidities, emergent or elective cholecystectomy, and perioperative outcomes. In addition, the SPARCS database includes all levels of medical institutions, which ignores the fact that different levels of hospitals and surgeons with different levels of experience and medical resources may impact the surgical outcomes. Therefore, there is not significant evidence to conclude the precise duration between PC and cholecystectomy yet, and more convincing evidence is required in the future.

**Table 1:** Summary of literature reviews on PC followed by cholecystectomy.

**Years**	**Authors**	**Study design**	**Database**	**Comparison**	**Results**
2009	Kim et al. (48)	Retrospective	Single medical institute	≦7 days (*n* = 35) vs. 14–39 days (*n* = 38)	≦7 days group had a shorter total hospital stay
2011	Han et al. (55)	Retrospective	Single medical institute	≦72 h (*n* = 21) vs. >72 h (*n* = 46)	≦72 h group had a prolonged operation time
2015	Jung et al. (56)	Retrospective	Single medical institute	≦10 days (*n* = 30) vs. >10 days (*n* = 44)	No differences among operation time, postoperative hospital stay, conversion to open cholecystectomy, or postoperative complications were observed
2016	Tanaka et al. (57)	Retrospective	Single medical institute	≦13days (*n* = 16) vs. >13 days (*n* = 47)	≦13 days group had more intraoperative blood loss
2017	Inoue et al. (58)	Retrospective	Single medical institute	≦9 days (*n* = 14) vs. >9 days (*n* = 53)	≦9 days group had a higher rate of postoperative complications and prolonged operation time
2019	Altieri et al. (59)	Retrospective	New York State SPARCS Database^*a*^	≦8 weeks (*n* = 1,211) vs. > 8 weeks (*n* = 1,787)	≦8 weeks group had a higher rate of complications and longer length of stay

a *SPARCS, Statewide Planning and Research Collaborative System*.

In summary, cholecystectomy following PC is a safe and effective combined therapy strategy for patients who cannot tolerate definitive surgery at the initial stage of AC. Cholecystectomy following PC can reduce the recurrence of biliary events and recurrent biliary event-related expenses and could be performed with low rates of postoperative complications and mortality.

### Acute Cholecystitis Patients Undergoing Percutaneous Cholecystostomy Without Interval Cholecystectomy

Although interval cholecystectomy has been recommended as a definitive treatment, many patients cannot tolerate the surgical risk or anesthesia risk due to the presence of multiple comorbidities and have no choice but to live with gallstones (29). Tokyo Guidelines 2018 recommended that for these patients with a CCI ≥ 4 or ASA-PS ≥ 3, conservative treatments should be considered without definitive surgery (10). For asymptomatic patients, the PC tube may be removed later with clinical surveillance. However, the PC tube may not always be removed smoothly, and some patients may still suffer from gallstone-related symptoms even after PC placement in the acute stage. As previously mentioned, up to 22–41% of AC patients may suffer from the recurrence of biliary events (12, 15, 20, 37). In addition, Bala et al. also identified two independent risk factors for permanent indwelling PC tubes: age > 75 years old and serum alkaline phosphatase level > 135 IU/L (60). For these symptomatic patients, the PC tube may remain in place for a long time. Some authors suggested that cholecystoscopy with cholecystolithotomy via a PC tube with a later attempt at PC removal may be a safe and effective management strategy for reducing recurrent biliary events (61–65).

In summary, the long-term use of a PC tube can be an alternative treatment without later interval cholecystectomy in AC patients who are unfit for surgery, even though these patients may experience a recurrence of biliary events. Therefore, patients and their caretakers should be well-educated on recurrent biliary events.

## PC-associated Complications and Care

### Complications

The reported incidence of PC-related complications varies from 2.5 to 69% (15, 20, 25, 38, 49, 66–68). Among all the complications, dislodgement of the cholecystostomy tube is the most common occurrence, which can account for more than half of all events in some publications (20, 27, 38, 66, 68). Apart from tube dislodgement, bile leakage is another common complication (12, 15, 49, 61, 68). Other events, such as bleeding, obstruction of the tube, infection, organ perforation, and mortality, have also been reported but are relatively rare (15, 20, 25, 33, 38, 61, 68). The management of complications varies and is usually individualized. There is scarce literature regarding management of PC related complications. Venkatanarasimha et al. published a detailed narrative review regarding the diagnosis and management for the complications of varietal kinds of biliary interventions, including both PC procedures and non-PC procedures, from the aspect of interventional radiologists (69). Therefore, we can only share our own experience regarding management of PC related complications. For patients with complete dislodgement of the PC tube, a full evaluation is required. After the patient is confirmed to be asymptomatic, he or she can be discharged without a repeat PC. For patients with suspected partial dislodgement of the PC tube, cholangiography can be utilized to confirm the position of the drainage tube. The decision to maintain or remove the drainage tube is made by physicians or radiologists based on the general condition of the patients. Among patients complicated with bile leakage which are usually symptomatic, antibiotics and image-guided drainage should be considered. Most patients complicated with minor bleeding can be managed conservatively. However, in patients with major bleeding, embolization with a coil or immediate laparotomy to stop the bleeding may be chosen. Regarding patients with suspected tubal obstruction, bedside irrigation and cholangiography can be arranged. If the examination indicates tubal obstruction, subsequent management, including reinsertion of the PC tube or emergent cholecystectomy, should be performed according to the clinical condition of the patient. For patients physically fit for surgery, cholecystectomy is recommended. However, if he or she refuses surgery or has multiple comorbidities, replacement of the PC tube is recommended.

### PC Tube Removal vs. PC Tube Maintenance

The issue of whether to remove or maintain PC tubes after the resolution of AC is rarely addressed or emphasized. Since there is no recommendation under the current guidelines, the policy on the removal or maintenance of PC tubes is still inconsistent (9, 10). Some authors believe that PC tubes should be preserved until surgery because removal is associated with the recurrence of cholecystitis and complicated with mortality, and this policy was applied in several medical institutes (38, 60, 61, 70). To prevent malfunction or obstruction of the drainage tube, routine replacements of the tube are necessary (14, 18). However, an increasing number of authors have proposed that the PC tube should be removed after the resolution of AC, which is supported by an increasing amount of evidence (12, 15, 33, 38, 44). Several findings support PC tube removal. First, the tract only requires 3–4 weeks to mature; after that, the mature tract will prevent bile from leaking into the intraperitoneal space, which means that the drainage tube can be safely removed (17, 71). Most studies were designed with PC tube removal at least 3 or 6 weeks after PC placement (14, 15, 29, 38, 61, 72). However, one systematic review, conducted by Macchini et al., revealed that the duration that the PC tube remains in place may not affect the clinical outcomes (73). Further research about the optimal timing of PC tube removal may be required. Second, prolonged indwelling of the PC tube was found to be a precipitating factor for recurrent biliary events in patients unfit for surgery (37). Third, removal of the PC tube had no impact on the clinical outcomes of patients with or without later interval cholecystectomy (24, 29, 32, 74). Regarding patients who were unfit for interval cholecystectomy, Cha et al. conducted a comparative analysis between the tube removal group and the tube maintenance group and showed no difference in recurrence (24). For patients undergoing interval surgery at a later date, one recent study indicated that AC may recur more frequently in the tube removal group, but subsequent cholecystectomy could be performed safely, even as emergent surgery (74). Based on the aforementioned evidence, PC tube removal seems more rational.

Prior to removing the PC tube, we suggest performing a clamping test. While there is no standardized clamping test, patients should be able to tolerate continuous clamping for at least 24–48 h (24, 45, 61). During the clamping period, there should be no symptoms and signs of recurrence; otherwise, the drainage tube cannot be removed. In summary, even though there are no guidelines regarding the removal or maintenance of PC tubes, more evidence seems to favor the decision to remove PC tubes.

### Cholangiography

**Figure 1:**
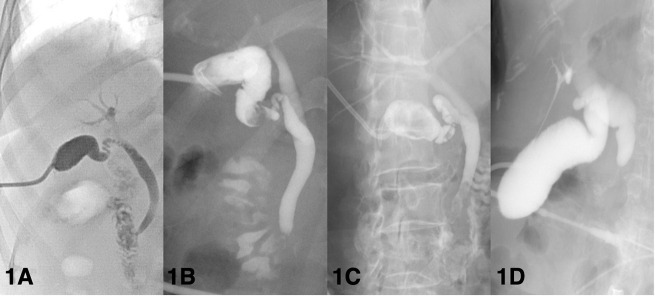
Various definitions of patent biliary tree according to cholangiography findings. **(A)** Patent biliary tree. Both the cystic duct and common bile duct are patent to the duodenum. **(B)** Gallstones at the gallbladder neck. Patent cystic duct and common bile duct. **(C)** Choledocholithiasis. Patent cystic duct. **(D)** Occluded distal common bile duct without opacification of the duodenum.

**Figure 2:**
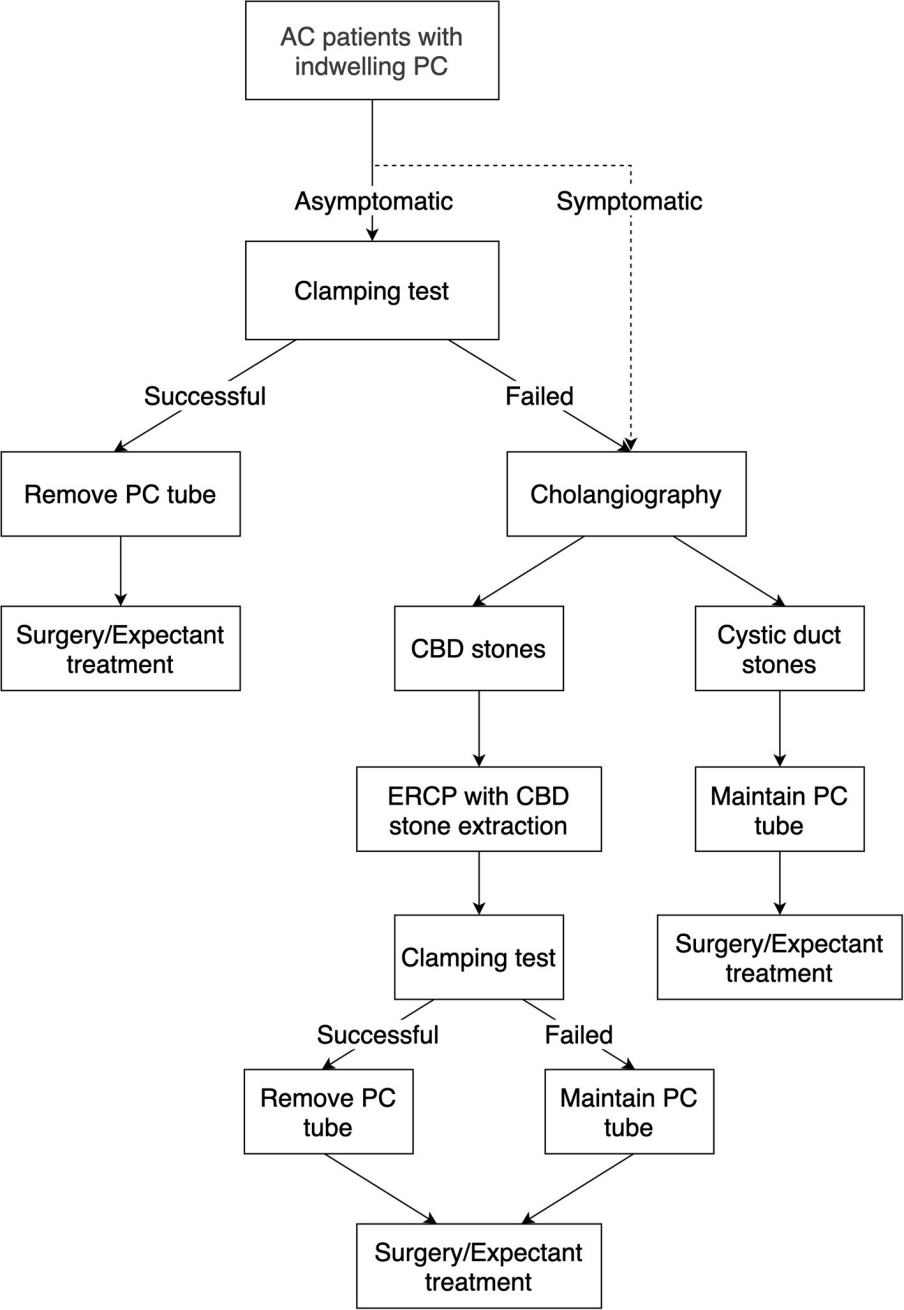
Proposed management algorithm for AC patients following PC placement.

Cholangiography via the PC tube can visualize the biliary tree, and the obstructed level of the biliary tree can be identified. After the patients are placed on the table in the supine position, the appropriate contrast medium was injected into the cholecystostomy tube to visualize the bile duct; after that, X-rays were taken. Typically, cholangiography is arranged at least 3 weeks after PC placement to confirm the patency of the biliary tree. The definitions of a patent biliary tree are heterogenous (Figure 1). Some physicians considered patency to be defined as no obstruction of cystic duct and common bile duct with illustration of duodenum, others considered patency to be defined as non-obstructed cystic ducts, which mainly focus on the outlet of gallbladder (15, 24, 32, 44, 61, 74). A patent bile duct is important because residual choledocholithiasis in the biliary tree is a potential risk factor for recurrent cholecystitis (12, 45). For AC patients with PC placement, cholangiography was commonly utilized during the follow-up period or when concerning the removal of PC tubes. Regarding symptomatic patients, such as those with a reduced amount of bile drainage, biliary colic, and suspicion of dislodgement, cholangiography is commonly applied (75). Regarding asymptomatic patients considering PC tube removal, most physicians performed cholangiography and clamping tests in combination, namely because both patency on the cholangiogram and tolerance to the clamping test were essential factors prior to removing the PC tube (24, 32, 38, 61). However, this concept has been challenged in the last 5 years. Our previous retrospective study showed no significant differences in perioperative complications, postoperative hospital stays, and emergent cholecystectomy between patients who underwent cholangiography and those who did not (74). Loftus et al. conducted a retrospective study focusing on patients who underwent routine surveillance cholangiography and patients who underwent on-demand cholangiography and revealed that patients who underwent on-demand cholangiography were associated with early PC tube removal and early cholecystectomy, but both groups had similar recurrence rates (76). Furthermore, Park et al. indicated that cholangiography was not associated with a lower recurrence rate, while the use of a clamping test was a protective factor associated with a lower incidence of recurrent AC (45). In our opinion, since the clamping test may lower the recurrence rate of AC after tube removal, applying the clamping test as a screening tool prior to cholangiography may be a novel approach in patients suitable for PC tube removal (Figure 2).

## Future Directions

The optimal timing of interval cholecystectomy is still one of the major issues for AC patients with PC placement. All published studies have been designed as comparative analyses comparing perioperative outcomes before and after a certain cut-off point; however, the results seemed to vary. Apart from an interval of 6–8 weeks suggested by most textbooks and clinical experts, no consensus has been reached (77, 78). We believe that more convincing evidence, such as data from randomized control studies and systemic reviews and more real-world data, is necessary to set future treatment guidelines.

Some authors mentioned that the introduction of the Tokyo Guidelines has increased the usage of PC, leading to fewer cholecystectomies and an increased incidence of recurrent biliary events in the past decade (79, 80). In addition, the medical expenses may also increase. Wang et al. indicated that the expenses associated with recurrent biliary events are 1.75 times greater than the expenses of patients who underwent definitive cholecystectomy (54). The primary function of PC is to de-escalate the inflammation or infection status of AC patients who cannot tolerate the surgical or anesthesia risk rather than limiting the chances of these patients to receive definitive surgery. It is crucial to identify patients who are unfit for surgery or fit for surgery followed by PC. Some authors have addressed the many aspects of heterogeneity among AC patients receiving PC, for instance, in disease severity, anesthesia risk, comorbidities, and survival time; thus, AC patients with PC placement who were fit for interval/elective surgery were essentially different from AC patients with PC placement who were unfit for interval/elective surgery (23, 35, 44, 60, 68). In addition, the latest Tokyo Guidelines 2018 has revised the algorithm for severe cholecystitis and have recommended that patients with a CCI of 4 or greater and ASA-PS of 3 or greater should receive expectant management; however, the efficacy of this classification has not been investigated (10). Future research on the efficacy of the latest classification and new objective factors that can distinguish patients who are fit or unfit for surgery are required.

## Conclusion

PC is a feasible, safe, and reliable intervention for AC patients who cannot tolerate immediate surgery due to the presence of severe comorbidities. The management of the PC tube is diverse and individualized. Some patients who are too ill to receive interval surgery can only live with gallstones, and others who fully recover from the acute phase of AC can receive definitive cholecystectomy in the future. While an interval of 6–8 weeks between PC and cholecystectomy is commonly applied, the optimal timing is still inconsistent. Regarding post-PC management, either maintaining or removing the PC tube is acceptable, but more evidence seems to support removal of the PC tube. Prior to removing the PC tube, the strategy of cholangiography combined the clamping test is commonly applied; nevertheless, the potential of the clamping test seems to be underestimated.

## Author Contributions

S-YW and Y-LH: idea for the article. Y-LH: literature research and figures and tables. C-MS, S-YW, Y-YJ, C-NY, T-SY, and J-TH: literature approval. Y-LH and S-YW: article drafting. C-MS, S-YW, C-YF, and C-HL: article revision. C-MS, Y-YJ, C-NY, T-SY, and J-TH: final approval. All authors contributed to the article and approved the submitted version.

## Conflict of Interest

The authors declare that the research was conducted in the absence of any commercial or financial relationships that could be construed as a potential conflict of interest.

## Abbreviations

PC: Percutaneous cholecystostomy
AC: Acute cholecystitis
PTGBD: Percutaneous transhepatic gallbladder drainage
LC: Laparoscopic cholecystectomy
WSES: World Society of Emergency Surgery
CCI: Charlson comorbidity index
ASA-PS: American Society of Anesthesiologists physical status
WBC: White blood cell
CRP: C-reactive protein.
